# Incremental Propensity Score Interventions: A Primer for Pharmacoepidemiologists

**DOI:** 10.1002/pds.70484

**Published:** 2026-09-24

**Authors:** Byeong Yeob Choi

**Affiliations:** ^1^ Department of Population Health Sciences Long School of Medicine, UT San Antonio Health Science Center San Antonio Texas USA

**Keywords:** abciximab, average treatment effect, incremental propensity score intervention, machine learning, uniform inference

## Abstract

**Background:**

In observational pharmacoepidemiology, estimating average treatment effects (ATEs) is often challenging due to a lack of practical positivity. In highly selective clinical settings, certain patients almost always or never receive treatment, causing ATE estimators to rely on unstable extrapolation. Incremental propensity score interventions (IPSIs) offer a stochastic alternative by shifting each patient's probability of treatment, providing a more clinically realistic framework that circumvents positivity violations.

**Methods:**

We illustrate the IPSI approach, including key identification results and inferential procedures. Using observational data from a cohort of 996 patients undergoing percutaneous coronary intervention (PCI), we evaluated the effect of shifting each patient's probability of receiving abciximab by a predetermined amount on six‐month mortality. Propensity scores (PSs) and outcome predictions were estimated using a machine learning ensemble (Super Learner) with 10‐fold sample splitting.

**Results:**

The ATE estimate suggested that abciximab administration reduced the 6‐month mortality risk by 5.9 percentage points compared with PCI alone (risk difference = −0.059, 95% CI: −0.104 to −0.015). However, the practical interpretability of the ATE estimate may be limited because it implicitly assumes that patients with a near‐certain probability of treatment could realistically be assigned to withhold abciximab. In contrast, shifting each patient's treatment propensity by odds ratios ranging from 0.1 to 10 showed that 6‐month mortality would be significantly reduced under a strong treatment policy promoting abciximab administration.

**Conclusions:**

IPSIs provide a robust and practical alternative to conventional causal inference methods in pharmacoepidemiology settings where treatment assignment is highly selective and the strict positivity is violated.

## Key Points

- In observational pharmacoepidemiology, conventional average treatment effect (ATE) estimation may be unreliable when practical positivity is violated because some patients are almost always or never treated.
- Incremental propensity score interventions (IPSIs) provide a clinically realistic alternative by estimating the effect of gradually shifting each patient's treatment probability.
- We applied IPSIs to evaluate the effect of abciximab on 6‐month mortality by modifying the odds of treatment assignment across a wide range of intervention scenarios.
- The estimated incremental effect curve showed that higher probabilities of abciximab administration were associated with lower 6‐month mortality risk.
- Statistically significant reductions, however, were observed only when the odds of treatment were increased substantially beyond observed clinical practice.

## Plain Language Summary

In observational pharmacoepidemiology studies, it is often difficult to estimate treatment effects reliably because some participants are much more likely than others to receive a treatment based on their demographic and clinical characteristics. In these situations, traditional causal inference methods that compare scenarios in which all individuals are either treated or untreated may rely on unrealistic assumptions. This study demonstrates the use of incremental propensity score interventions (IPSIs), a newer causal inference approach that evaluates more realistic changes in treatment practice. Instead of assuming that treatment can be administered or withheld across all patients, IPSIs examine what would happen if the probability of receiving treatment were gradually increased or decreased for each participant.

## 1 Introduction

In pharmacoepidemiology, causal questions are frequently formulated in terms of average treatment effects (ATEs), which compare outcomes under hypothetical interventions in which all individuals in a population receive treatment versus no individuals receive treatment. Under standard identification assumptions (e.g., exchangeability and positivity), ATEs can be estimated using four classical propensity score (PS) methods: matching, stratification, inverse probability of treatment weighting (IPTW), and covariate adjustment [1].

One important challenge in observational pharmacoepidemiology is the lack of practical positivity [2]. In clinical settings, treatment decisions are often highly selective according to disease severity, comorbidities, and physician preference. Consequently, some patients may almost always receive treatment, whereas others may never receive it. In such settings, estimation of the ATE may rely heavily on extrapolation into regions with limited covariate overlap, complicating interpretation and potentially increasing estimator instability.

Positivity violations can induce severe bias across all causal effect estimators [3]. To diagnose this, the parametric bootstrap provides a practical estimate of bias arising from positivity violations and near‐violations [4]. To address identified violations, researchers can (1) alter the projection function to weaken the needed positivity assumption [5, 6], (2) trim problematic covariates from the adjustment set, (3) trim extreme PSs [7], or (4) modify the intervention of interest [8, 9, 10]. This paper focuses on the fourth strategy via incremental propensity score interventions (IPSIs) [10], which bypass strict positivity constraints.

The IPSIs, introduced by Kennedy [10], provide an alternative causal framework that could be more appropriate for pharmacoepidemiologic settings characterized by positivity concerns. Rather than deterministically assigning treatment to all individuals, IPSIs bypass positivity violations by quantifying the effect of shifting each patient's baseline probability of treatment by a user‐specified amount. Such interventions may better represent policy changes and guideline modifications in clinical practice.

To illustrate the practical value of IPSIs in pharmacoepidemiology, we evaluate the causal effect of incremental changes in abciximab administration on 6‐month mortality among patients undergoing percutaneous coronary intervention (PCI) [11]. Abciximab utilization is heavily confounded by acute clinical presentation, making traditional deterministic interventions prone to positivity violations. Utilizing Kennedy's IPSI framework, we demonstrate how shifting each patient's baseline propensity to receive abciximab provides robust, policy‐relevant causal estimates of realistic interventions that modify prescribing practices.

## 2 Methods

### 2.1 Conventional PS Analysis

Identifying and estimating the ATE is based on the potential outcomes framework, where $Y^{1}$ denotes the potential outcome if a patient receives treatment and $Y^{0}$ denotes the potential outcome if a patient does not receive treatment. Under the consistency assumption, the observed outcome Y equals $Y^{1}$ for patients who actually received treatment $\left(A=1\right)$ and equals $Y^{0}$ for patients who did not receive treatment $\left(A=0\right)$. We define the PS as the probability of receiving treatment conditional on observed covariates X: $\pi \left(X\right)=\mathrm{Pr}\left(A=1|X\right)$ [12].

The ATE is typically estimated using four PS methods [1]. PS matching forms pairs or sets of treated and untreated individuals who share similar estimated PSs [13]. PS stratification ranks individuals according to their estimated PSs and divides them into mutually exclusive strata [14]. IPTW assigns each individual a weight equal to the inverse of the probability of receiving the treatment they actually received, thereby creating a pseudo‐population in which measured baseline covariates are independent of treatment assignment [5]. Finally, PS covariate adjustment incorporates the estimated PS directly as a covariate in the outcome model [15].

Although these methods are effective for controlling measured confounding, their performance can deteriorate substantially when the positivity assumption is violated. In IPTW, PSs near 0 or 1 produce extreme weights, leading to unstable estimates and increased sensitivity to model misspecification [16, 17]. In PS matching, limited treatment overlap forces the exclusion of unmatched individuals, thereby changing the target estimand [13]. In subclassification, extreme PSs can produce strata containing very few treated or untreated individuals, making within‐stratum treatment effect estimation unreliable. PS covariate adjustment relies on extrapolation in regions of the covariate space where one treatment group is poorly represented.

### 2.2 Augmented Inverse Probability Weighting (AIPW)

Outcome regression models can be integrated into the PS methods (matching, subclassification, and IPTW) to reduce bias arising from PS model misspecification and improve estimator precision [15, 18]. In particular, AIPW combines outcome predictions with standard IPTW weights. AIPW is doubly robust, meaning the treatment effect estimator remains consistent if either the PS model or the outcome model is correctly specified [19]. When both models are correctly specified, the estimator achieves semiparametric efficiency [20]. Furthermore, its asymptotic distribution can be characterized using its influence function, which allows the variance to be consistently estimated from the sample variance of the estimated influence function [21, 22]. However, when positivity is violated, consistency relies entirely on the correct specification of the outcome model [3].

### 2.3 IPSI

Instead of targeting the ATE, we may be interested in estimating the effect of a hypothetical intervention that changes each patient's probability of receiving abciximab. Specifically, rather than setting treatment to everyone or no one, we consider interventions that shift each patient's PS by a fixed multiplicative factor on the odds scale.

For example, suppose a patient has a propensity for receiving abciximab of $\pi \left(X\right)=0.2$ under the current treatment policy. Under a hypothetical intervention, suppose that this propensity is increased to $q\left(X\right)=0.5$. The corresponding change in the odds of treatment is:

$$\frac{0.5/\left(1-0.5\right)}{0.2/\left(1-0.2\right)}=4.$$

Thus, the intervention multiplies the patient's odds of receiving abciximab by δ=4.

Incremental PS interventions generalize this idea by multiplying the odds of treatment for every patient by a common factor, δ. The resulting estimand is the 6‐month mortality risk that would be observed if each patient's odds of receiving abciximab were multiplied by δ. For a given value of δ, the shifted PS is:

(1) $$q\left(\delta ,X\right)=\frac{\delta \pi \left(X\right)}{\delta \pi \left(X\right)+\left\{1-\pi \left(X\right)\right\}}.$$

By construction:

$$\delta =\frac{q\left(\delta ,X\right)/\left\{1-q\left(\delta ,X\right)\right\}}{\pi \left(X\right)/\left\{1-\pi \left(X\right)\right\}}.$$

Therefore, δ is the odds ratio comparing the shifted and original PSs. Consequently, δ represents the multiplicative change in each patient's odds of receiving abciximab under the hypothetical intervention. Values of δ>1 increases the odds of treatment, whereas values of δ<1 decreases them.

Unlike traditional PS methods, the IPSI framework shifts the odds of treatment relative to an individual's baseline probability rather than evaluating a deterministic assignment. Consequently, it relaxes the strict positivity assumption by not requiring that every individual have a non‐zero probability of experiencing both treatment states. As demonstrated by Equation (1): boundary PSs of 0 or 1 remain unshifted since $q\left(\delta ,X\right)=0$ when $\pi \left(X\right)=0$ and $q\left(\delta ,X\right)=1$ when $\pi \left(X\right)=1$. This ensures that structural zero or one probabilities are preserved rather than artificially shifted toward the opposite extreme. In contrast, the ATE framework assumes counterfactual outcomes even for individuals who cannot realistically receive one of the treatment options.

To identify IPSI effects, it is sufficient to assume that $Y=Y^{a}$ when $A=a\in \left\{0,1\right\}$ for consistency, and that $Y^{a}$ is independent of A conditional on X for exchangeability. We express the potential outcome under a stochastic intervention as $Y^{Q\left(\delta \right)}$, where Q represents draws from the conditional distribution $q\left(\delta ,X\right)$, and is upper‐case because the intervention is stochastic [10].

Following Corollary 1 of Kennedy [10], the mean counterfactual outcome $\psi \left(\delta \right)=E\left[Y^{Q\left(\delta \right)}\right]$ under the IPSI is identified by

(2) $$\begin{array}{c} \psi \left(\delta \right)=E\left[\frac{\delta \pi \left(X\right)\mu \left(X,1\right)+\left\{1-\pi \left(X\right)\right\}\mu \left(X,0\right)}{\delta \pi \left(X\right)+\left\{1-\pi \left(X\right)\right\}}\right], \end{array}$$

where $\mu \left(x,a\right)=E\left[Y|X=x,A=a\right]$. Equation (2) shows that the IPSI effect is a weighted average of the regression functions $\mu \left(X,1\right)$ and $\mu \left(X,0\right)$, where the weight on $\mu \left(X,1\right)$ is the shifted PS in Equation (1). An important special case arises when δ=1: $\psi \left(\delta \right)$ is the original outcome mean, $\psi \left(1\right)=E\left[Y\right]$. There are two extreme cases of $\psi \left(\delta \right)$: when δ→0 or δ→∞, $\psi \left(\delta \right)$ approaches $E\left[Y^{0}\right]$ or $E\left[Y^{1}\right]$ if positivity holds.

### 2.4 Uniform Inference on an Incremental Effect Curve

While a pointwise confidence interval based on classical asymptotic theory allows for inference on the IPSI at a fixed value of δ, evaluating an incremental effect curve $\psi \left(\delta \right)$ over a range of δ values provides a more comprehensive assessment. Specifically, it allows researchers to determine whether IPSI effects achieve statistical significance across a chosen range of δ. To achieve this, the uniform inference technique described by Kennedy [10] is required to construct a uniform confidence band that covers the entire incremental effect curve with a prespecified probability.

Let $D=\left[\delta_{l},\delta_{u}\right]$ denote the chosen range of δ, where $\delta_{l}$ and $\delta_{u}$ are the lower and upper bounds of the range. Over D, we can test the null hypothesis of no IPSI effect at significance level of α

$$H_{0}:\psi \left(\delta \right)=E\left[Y\right]\;\text{for}\;\mathrm{all}\;\delta \in D,$$

by assessing whether the $\left(1-\alpha \right)$ uniform confidence band contains the horizontal line corresponding to $E\left[Y\right]$ across the entire range of D. This null hypothesis implies that the potential mean outcome under the shifted PS distribution does not differ from the observed mean outcome.

Estimation of $\psi \left(\delta \right)$ involves both the PS and outcome models. Kennedy proposed using machine learning methods, such as Super Learner [23], to mitigate the impact of model misspecification, along with sample splitting [24] to reduce bias arising from the double use of data. Super Learner constructs an optimal weighted combination of multiple candidate learners, where the weights are selected to maximize predictive performance. This approach is, particularly, useful for capturing complex relationships between confounders and either treatment assignment or outcomes [25, 26]. Sample splitting further enables statistically valid causal inference with flexible machine learning methods by separating the data used for model training from the data used to estimate nusaince paramters.

Based on the estimated PSs and outcome predictions, we constructed a 95% uniform confidence band around the incremental effect curve [10]. The critical value is determined such that the probability that the maximum absolute value of the normalized statistics over the range D is less than the critical value equals 0.95. In general, this critival value exceeds 1.96, the conventional critical value for a 95% confidence interval. The npcausal R package [27] was used to implement the IPSI and AIPW methods.

## 3 Application

We applied the proposed method to evaluate the effect of abciximab on 6‐month mortality among patients undergoing PCI. Abciximab is an adjunctive pharmacotherapy used to reduce periprocedural ischemic risk. Using observational data originally analyzed by Kereiakes et al. [11], we studied a cohort of 996 patients, including 698 who received abciximab and 298 who underwent PCI alone, to estimate the IPSI of abciximab on 6‐month mortality.

The dataset included seven patient and procedural characteristics: three continuous and four binary variables. The continuous variables were height (108–196 cm), left ventricular ejection fraction (LVEF, 0%–90%), and the number of vessels involved in the initial PCI (NVIP, 0–5). The binary variables included coronary stent deployment, female sex, diabetes mellitus diagnosis (DMD), and acute myocardial infarction (AMI) within the preceding 7 days.

In this application, stochastic interventions may be more appropriate than deterministic interventions. Certain combinations of demographic and clinical characteristics were associated with a very high probability of receiving abciximab, making hypothetical interventions that prohibit treatment assignment clinically unrealistic. For example, male patients with stent deployment and recent AMI but without DMD had a 94% probability of receiving abciximab. IPSI provides a more realistic framework for handling patients with PSs of abciximab close to 1 by shifting their treatment probabilities rather than forcing them to zero.

Using IPSI, we quantified 6‐month mortality that would be observed if each patient's propensity for receiving abciximab were modified by odd ratios ranging from 0.1 to 10. This range was selected to cover intervention scenarios in which the odds of treatment were either decreased or increased by a factor of 10 on the multiplicative scale.

PSs were estimated using Super Learner with the following base learners: mean model, logistic regression with main effects, logistic regression with main and interaction effects, generalized additive models [28], recursive partitioning and regression trees [29], random forests [30], and multivariate adaptive regression splines [31]. In addition, we used 10‐fold sample splitting to avoid potential overfitting. The Super Learner‐estimated PSs were compared with those obtained from conventional logistic regression including only main effects. The two approaches produced similar PSs with a correlation coefficient of 0.88, suggesting that the nonlinearity and nonadditivity in the covariate effects may not be strong. However, the Super Learner tended to produce smaller PSs than logistic regression. Outcome predictions were performed using the same approach as PS estimation, employing the Super Learner with the same set of base learners and 10‐fold sample splitting.

**FIGURE 1:**
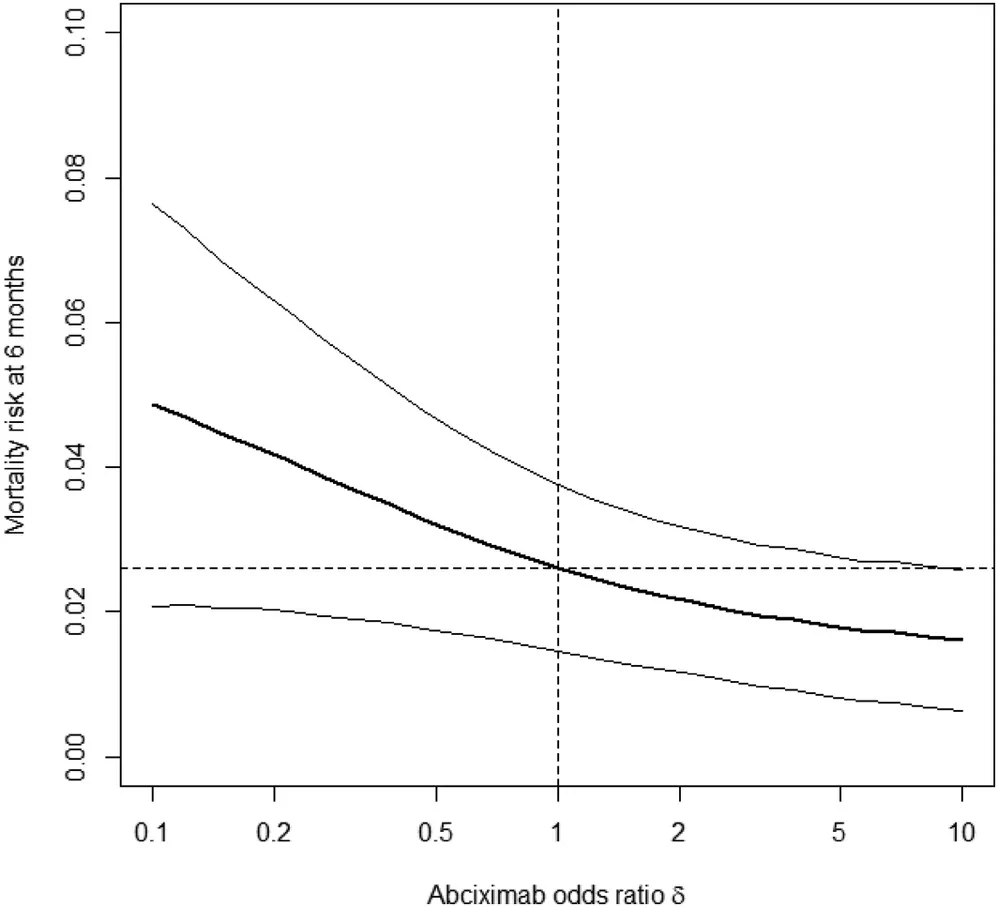
Bold line indicates the estimated 6‐month mortality risk under incremental shifts in the odds of abciximab administration by a factor δ. The surrounding lines represent the 95% uniform confidence band for the incremental effect curve.

Figure 1 presents the estimated 6‐month mortality risk under IPSIs that modify the odds of receiving abciximab among patients undergoing PCI, indexed by the shift parameter δ. The horizontal axis represents the multiplicative change in the odds of treatment assignment, where δ=1 corresponds to the observed treatment distribution, values less than 1 represent interventions that reduce the odds of abciximab use, and values greater than 1 represent interventions that increase the odds of treatment.

The incremental effect curve in Figure 1 indicates that the estimated mortality risk decreases as δ increases, suggesting that increasing the probability of abciximab administration is associated with lower 6‐month mortality risk. The estimated critical value for the confidence band was 2.32. However, the band covers the observed mortality risk of 0.026 across most values of δ. The upper bound of the confidence band falls slightly below the observed mortality risk only when δ approaches 10, indicating only marginal evidence against the null hypothesis. These findings suggest that the incremental effect of abciximab on reducing 6‐month mortality would not be significant unless the odds of treatment were increased substantially relative to observed clinical practice.

The AIPW estimator targeted the ATE, defined as the difference in 6‐month mortality rates that would have been observed if all patients had received versus not received abciximab. It was computed using PSs and outcome predictions estimated via Super Learner based on the same set of base learners as the IPSI along with 10‐fold sample splitting, and its variance was estimated using the efficient influence function [20, 24]. In contrast to the IPSI curve, the AIPW estimate suggested a clinically meaningful and statistically significant benefit of abciximab with a risk difference of −0.059 (95% CI: −0.104 to −0.015; *p* = 0.008).

## 4 Conclusion

The IPSI estimand addresses key theoretical and practical limitations of deterministic causal estimands, such as the ATE, by shifting individual treatment propensities rather than assigning a fixed exposure state. Crucially, this approach circumvents strict positivity assumptions because it does not require every individual to have a nonzero probability of receiving all treatment options. Consequently, at the policy level, the IPSI framework transforms causal evaluation from unrealistic treatment mandates toward actionable, population‐level strategies. Clinically, it models interventions such as updated practice guidelines or reimbursement changes that incrementally shift treatment patterns.

The IPSI shares features with both PS methods and Robin's *g*‐formula [32], while differing from each in important ways. The IPSI uses the PS to modify treatment probabilities under hypothetical interventions. However, unlike conventional PS methods, the PS is not used to control for confounding. Nevertheless, as with standard PS approaches, misspecification of the PS model can bias IPSI effect estimates because the intervention is defined through the PS. Instead, confounding adjustment is achieved through the outcome regression functions. In this sense, IPSI can be viewed as a special case of *g*‐formula, in which outcome regressions are evaluated over the range of treatment probabilities where positivity holds.

There are a few limitations of the IPSI approach thar are worth noting. First, by virtue of their dependence on the observational treatment process, incremental intervention effects play a more descriptive role than prescriptive compared to other approaches [10]. Specifically, they describe the potential consequences of realistic, marginal shifts in the propensities of receiving treatment, but will likely be less useful for informing specific treatment decisions [10]. Second, translating the shift parameter δ into a concrete, real‐world policy remains a challenge. While we can numerically model a twofold increase in the odds of treatment, designing a physical intervention to achieve that probability shift is difficult in practice. Finally, even though the IPSI approach does not require positivity for identifiability, it still relies on other fundamental assumptions, including exchangeability, no interference, and consistency [33].

## Author Contributions

B.Y.C. conceived the research question, conducted analyses, and wrote the manuscript.

## Funding

This research was supported in part by the National Cancer Institute for the Mays Cancer Center (P30CA054174) at UT San Antonio Health Science Center.

## Ethics Statement

The author has nothing to report.

## Conflicts of Interest

The author declares no conflicts of interest.

## Data Availability

The data that support the findings of this study are openly available in lindner dataset at https://r‐packages.io/datasets/lindner.
